# Intestinal Parasites Infection in Children with Cancer in Ahvaz, Southwest Iran

**DOI:** 10.1155/2020/8839740

**Published:** 2020-12-24

**Authors:** Roya Salehi Kahyesh, Arash Alghasi, Shekoufe Haddadi, Asaad Sharhani

**Affiliations:** ^1^Thalassemia and Hemoglobinopathy Research Center, Health Research Institute, Ahvaz Jundishapur University of Medical Sciences, Ahvaz, Iran; ^2^Department of Pediatrics, School of Medical, Ahvaz Jundishapur University of Medical Sciences, Ahvaz, Iran; ^3^Department of Epidemiology, School of Public Health, Shahid Beheshti University of Medical Sciences, Tehran, Iran

## Abstract

**Background:**

Infection with intestinal parasites is widespread worldwide, especially in developing countries. Intestinal parasites are known as one of the leading causes of diarrhea in both immunocompetent and immunocompromised subjects, but cancer patients are highly susceptible to contamination, and it can be deadly for them. This study aimed to estimate the prevalence of intestinal parasites in immunocompromised patients in Ahvaz. *Material and Methods*. In this descriptive cross-sectional pilot case-control study, fecal samples were collected from 52 children with malignancies hospitalized in Baqaei2 hospital in Ahvaz. A questionnaire including demographic information, type of cancer, type of gastrointestinal symptoms, and laboratory diagnosis was completed for each patient. The collected specimens were examined by direct smear, Logul staining, and concentration.

**Result:**

The 52 stool samples were collected, 46% were female and 54% male. The age range of children enrolled in the study was from 4 months to 16 years. Of these stool samples, 38.38% were infected with a variety of parasitic intestinal infections (helminths and protozoa). In this study, protozoan parasites, *Blastocystis* (23%), *Chilomastix mesnili* (1.92%), *Endolimax nana* (7.7%), and *Entamoeba coli* (1.92%), and helminth infection, *Strongyloides stercoralis* (3.84%), were observed and statistical analysis showed that there was a significant relationship between gastrointestinal symptoms and parasitic infection in children with cancer.

**Conclusion:**

*Blastocystis* and *Endolimax nana* are the most prevalent gastrointestinal parasitic protozoans that infect individuals admitted to Baqaei2 Hospital of Ahvaz, Iran. Since parasitic intestinal infections in immunocompromised patients lead to fatal diarrhea, children with parasitic infections must be carefully identified and treated.

## 1. Introduction

Despite sanitation and hygiene education in recent decades, intestinal parasites infections continue to be characterized as a significant cause of morbidity worldwide, and according to World Health Organization (WHO) statistics, more than a quarter of the world's population, mostly in developing countries, are infected [1–3]. Intestinal parasites have been described as opportunistic infections in immunocompromised patients, such as HIV/AIDS patients, organ transplant recipients, hemodialysis patients (HD), and cancer patients, and cause long-term and sometimes fatal diarrhea compared to healthy individuals [4]. The prevalence of these parasites varies depending on environmental (especially in tropical regions such as Iran), demographic, socio-economic, political, physiological, and immunological factors, and transmission occurs via direct person-to-person, animal-to-human, animal-to-animal, or indirectly by water, food, and possibly via air [5]. In developing countries, lack of access to healthcare and malnutrition increases the susceptibility to these infections [6]. According to researchers, the incidence of cancer is on the rise today, so anticancer drugs and bone marrow transplants lead to immune deficiencies and increased prevalence of various infections, especially in children [7]. The most common parasitic infections that affect cancer patients are caused by *Giardia lamblia*, *Cryptosporidium parvum*, *Cyclospora cayetanensis*, *Entamoeba histolytica*, and *Strongyloides stercoralis* [8–10]. Although many studies have investigated the prevalence of intestinal parasites in immunocompromised patients, there are very few studies on cancer patients. Therefore, this study aimed to evaluate the frequency of intestinal parasites in cancer patients in the south of Iran.

## 2. Material and Methods

The subjects were recruited from the oncology department of Baqaei2 Hospital of Ahvaz during six-month duration (from April to October 2019). Fifty-two stool samples were collected from patients between 4 months and 16 years of age with malignancies and underwent chemotherapy. At first, via a standardized questionnaire, all needed information included demographic information, kind of cancer, kind of gastrointestinal symptoms (such as stomachache, cramps, nausea, vomit, and diarrhea), and the appearance of stool (color and consistency), and stool test (three separate stool samples) results were registered. We prepared one direct saline smear with Lugol's iodine staining and examined by a light microscope at 40x magnification. Also, samples were screened using the formalin-ether condensation method and the results of a person's experiments registered on the questionnaire. Finally, the data were analyzed using the Statistical Package for Social Sciences (SPSS) software and chi-square test for quality variables.

## 3. Results

In this study, 52 stool specimens were obtained from children with cancer. 53.8% of patients were male, and 46.2% were female. The age range of patients was between 4 months and 16 years and classified into four age groups (1 year ≤, 1–5 years, 6–10 years, and 11–16 years) (Table 1).

The results showed that there was no statistically significant difference between sex, age, and intestinal parasites infection. The prevalence rate of intestinal parasites (helminth and protozoa) was 38.38% in children with different malignancies Table 2.

The results reveal that among 52 children with cancer and infection of intestinal parasites, there is only a significant relationship between *Blastocystis* infection and gastrointestinal symptoms. Of the 11 patients infected with *Blastocystis*, six patients (11.5 percent) had gastrointestinal symptoms, including stomachache, cramps, nausea, vomit, and diarrhea, and a significant relationship was observed. Regarding the type of malignancy and parasite reported, the highest rate of parasitic intestinal infection was in children with ALL.  Table 3 shows the frequency of intestinal parasites and the type of malignancy in children with cancer.

## 4. Discussion

Every year, eight million new cases of cancer are diagnosed worldwide, which is an increase of almost 40% over the last 20 years. Due to the use of chemotherapy drugs and followed by a decrease in the number of *T*  cells, the cancer patient's immune system is suppressed, so these patients are more susceptible to opportunistic pathogens such as bacteria, viruses, and fungal and particularly opportunistic parasites [11], and it is difficult to eradicate them since they result in severe and disseminated disease rather than localized infection. Therefore, early diagnosis, treatment, and control of the disease in immunocompromised patients are essential [12, 13].

Intestinal parasitic infections caused by intestinal helminths and protozoa are among the most common human infections endemic throughout the world. Protozoan parasites being single-celled can rapidly multiply inside the body leading to the development of a severe infection. Most of the protozoan infections tend to be asymptomatic. However, the common symptoms associated with it include abdominal discomfort, vomiting, and dysentery. Children, especially those who are involved with cancer, are the primary victims of gastrointestinal protozoan parasites [14]. Intestinal parasitic infections have been reported in different parts of Iran, and the most common infections were *Giardia lamblia* and *B. hominis* [15–17]. According to the previous studies, high prevalence of different intestinal parasitic infections was reported from Iranian HIV/AIDS patients [18, 19].

Zabolinejad et al. studied the prevalence of intestinal parasites in lymphohematopoietic malignancy children in Mashhad, Iran. In this study, 35.9% of patients had parasitic infections: *G. lamblia* 18%, *Entamoeba coli* 6.7%, *Blastocystis hominis* 5.6%, *Iodamoeba butschlii* 2.2%, *Chilomastix mesnili* 1.1%, *Hymenolepis nana* 1.1%, and *Enterobius vermicularis* 1.1% [20] and this in agreement with our finding. In the same study that was conducted by Mohammadi and his colleagues, the frequency of parasitic intestinal infections was studied in cancer patients of northwest Iran. The overall frequency was 10%, and these intestinal parasites were *Cryptosporidium* spp. *Oocyst* 4%, *Blastocystis hominis* 3%, *Giardia lamblia* 2%, and *Taenia* spp. 1% [21]. In the present study, the most common parasite was *Blastocystis* (21%), followed by *Endolimax nana* (7.7%). Then, *Chilomastix mesnili*, *Entamoeba coli*, *Strongyloides stercoralisi*, and *Enterobius vermicularis* were observed with a frequency of 1%. Also, the highest rate of parasitic infection was observed in patients with acute lymphoid leukemia (ALL) (13.5%), neuroblastoma (9.6%), and acute myeloid leukemia (AML) (5.8%), respectively. Parasitic infections are accompanied by loss of weight, anorexia, malabsorption syndrome, and in some cases, fever and abdominal pain in immunocompromised individuals. However, it is important to point out that parasites such as *Strongyloides stercoralis* may disseminate to other organs such as the bronchia, bile, and liver ducts, and producing symptomatology specific to the organ affected [22]; so, the diagnosis of different types of parasites in infected individuals will be so valuable. In this regard, Khaleel et al. also stated that enteric protozoan infection was 60% among children with cancers receiving chemotherapy and reported four protozoan species among patients: *G. lamblia* (24%), *Cryptosporidium parvum* (19%), *E. histolytica/E. dispar* (5%), and *B. species* (5%). The highest infection rate was found among children with lymphoma (77.27%), followed by 62.26% and 40% among patients with leukemia and solid tumors, respectively [23]. Rasti et al. detected parasitic intestinal infections among 25% of HIV/AIDS patients and 12.0% in renal transplant recipients, so these results showed a high prevalence of parasitic intestinal infections in immunocompromised patients [24]. Another study among leukemic patients with diarrhea revealed that the infection rate of intestinal parasitic infections was 9.9% in İzmir, Turkey [25]. Esteghamati and his colleagues found the high rate of infection with *Blastocystis hominis* in cancer patients, especially colorectal cancer patients (22.3%). They also showed that Cryptosporidium spp. was the primary cause of parasitic intestinal infection in patients with organ transplant (20%) compared to primary immunodeficiency patients (1.25%) [26]. As the previous studies have shown, our results have also shown that children affected by malignancies and under treatment by chemotherapy are highly prone to parasitic infections, and it could be due to their effects on immunity mechanisms that include a mucosal membrane, skin, quantitative and qualitative defect of granulocytes, and macrophages. So, regarding the fact that parasitic infections can be had irreparable effects on cancer patients, it should be considered during the diagnosis of cancer in order to treat and prevent their complications.

## Figures and Tables

**Table 1:** Demographic specification of patients and infection rate of intestinal parasites in cancerous children.

*Infection rate of intestinal parasites*		*Total*	*Number/percent*	*Male*	*Gender*
(19.2%)/10	*Male*	52	53.8%/(28)		
(11.5%)/6	*Female*		46.2%/(24)	*Female*	
3.8%/(2)	*Age groups*		15.4%/(8)	*Age groups*	
	1≤			1≤	
3.8%/(2)	1–5		34.6%/(18)	1–5	
11.5%/(6)	6–10		23%/(12)	6–10	
9.6%/(5)	11–16		27%/(14)	11–16	

**Table 2:** The frequency of intestinal parasites (helminth and protozoa) in children with different malignancies.

	Parasite	Number	Percent
Protozoa infection	*Blastocystis*	11	21
	*Chilomastix mesnili*	1	1.92
	*Endolimax nana*	4	7.7

Helminth infection	*Entamoeba coli*	^1^	1.92
	*Strongyloides stercoralis*	1	1.92
	*Enterobius vermicularis*	1	1.92

**Table 3:** The frequency of intestinal parasites and the type of malignancy in children with cancer.

	Type of malignancy	Parasite/numbers	(%) infection
1	*Blastocystis*/3	Neuroblastoma	9.6
	*Strongyloides* stercoralis/1		
	*Endolimax nana*/1		

2	*Blastocystis*/2	AML	5.8
	*Endolimax nana*/1		

3	*Blastocystis*/2	ALL	13.5
	*Chilomastix mesnili*/1		
	*Endolimax nana*/1		
	*Entamoeba coli*/1		
	*Enterobius vermicularis*/1		

4	*Blastocystis*/1	Ewing sarcoma	1.92
5	*Blastocystis*/1	HLH	1.92
6	*Blastocystis*/1	Osteosarcoma	1.92
7	*Blastocystis*/1	Neurofibromatosis	1.92

## Data Availability

All data used to support the findings of this study are included within the article.
